# Disentangling detrimental sand fly-mite interactions in a closed laboratory sand fly colony: implications for vector-borne disease studies and guidelines for overcoming severe mite infestations

**DOI:** 10.1186/s13071-023-06074-8

**Published:** 2024-01-05

**Authors:** Chukwunonso O. Nzelu, Claudio Meneses, Christina Bowhay, Iliano V. Coutinho-Abreu, Emily Bennett, Somayeh Bahrami, Brian Bonilla, Shaden Kamhawi, Jesus G. Valenzuela, Nathan C. Peters

**Affiliations:** 1 Department of Microbiology, Immunology, and Infectious Diseases, Snyder Institute for Chronic Diseases, Cumming School of Medicine, University of Calgary, Calgary, AB Canada; 2 Faculty of Veterinary Medicine, University of Calgary, Calgary, AB Canada; 3 Laboratory of Malaria and Vector Research, National Institute of Allergy and Infectious Diseases, National Institutes of Health, Rockville, MD USA; 4 Division of Biological Sciences, University of California, San Diego, USA; 5 Department of Parasitology, Faculty of Veterinary Medicine, Shahid Chamran University of Ahvaz, Ahvaz, Iran

**Keywords:** Laboratory colony, Sand fly, Mites, Leishmaniasis, Arthropod vectors

## Abstract

**Background:**

Vector sand fly colonies are a critical component of studies aimed at improving the understanding of the neglected tropical disease leishmaniasis and alleviating its global impact. However, among laboratory-colonized arthropod vectors of infectious diseases, the labor-intensive nature of sand fly rearing coupled with the low number of colonies worldwide has generally discouraged the widespread use of sand flies in laboratory settings. Among the different factors associated with the low productivity of sand fly colonies, mite infestations are a significant factor. Sand fly colonies are prone to infestation by mites, and the physical interactions between sand flies and mites and metabolites have a negative impact on sand fly larval development.

**Methods:**

Mites were collected from sand fly larval rearing pots and morphologically identified using taxonomic keys. Upon identification, they were photographed with a scanning electron microscope. Several mite control measures were adopted in two different laboratories, one at the Laboratory of Malaria and Vector Research, National Institute of Allergy and Infectious Diseases-National Institutes of Health (Rockville, MD, USA), and the other at the University of Calgary (Calgary, AB, Canada).

**Results:**

The mite species associated with sand fly colonies in the two laboratories were morphologically identified as *Tyrophagus* sp. and *Stratiolaelaps scimitus*. While complete eradication of mites in sand fly colonies is considered unrealistic, drastically reducing their population has been associated with higher sand fly productivity.

**Conclusions:**

We report a case of detrimental interaction between sand flies and *Tyrophagus* sp. and *S. scimitus* in a closed laboratory sand fly colony, discuss their impact on sand fly production and provide guidelines for limiting the mite population size in a closed laboratory colony leading to improved sand fly yields.

**Graphical abstract:**

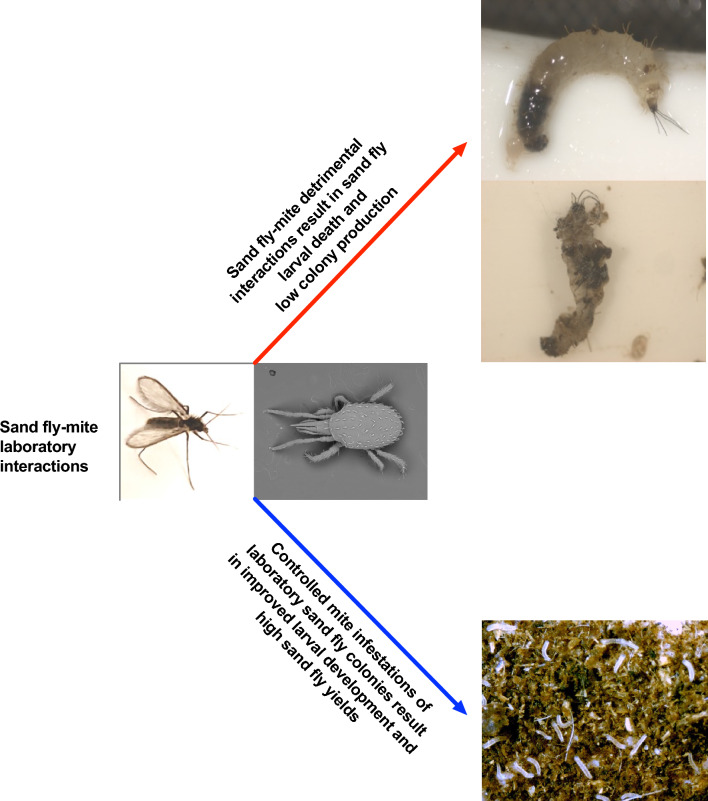

**Supplementary Information:**

The online version contains supplementary material available at 10.1186/s13071-023-06074-8.

## Background

Blood-sucking arthropods transmit a variety of infectious pathogens from infected human or animal hosts to naive uninfected human hosts and thus play a central role in the epidemiology of vector-transmitted diseases [1]. The establishment and maintenance of laboratory colonies of hematophagous arthropods are valuable for studies on vector potential, pathogen life-cycle, the impact of vector-mediated transmission on disease outcome and vaccine efficacy [2–5]. Some arthropod vectors have been successfully colonized in the laboratory for research purposes, such as mosquitoes, sand flies, triatomine bugs and ticks [6–9].

Sand flies are the vectors of leishmaniases, and approximately 1000 species have been recorded in six major genera: *Phlebotomus* (13 subgenera), *Sergentomyia* (10 subgenera) and *Chinius* (4 species) in the Old World, and *Lutzomyia* (26 subgenera and groups), *Brumptomyia* (24 species) and *Warileya* (6 species) in the New World [10–12]. However, only species belonging to the genera *Phlebotomus* and *Lutzomyia* are the putative vectors of *Leishmania* parasites [10, 13–15]. Unlike mosquitoes, sand flies do not have an aquatic stage in their life-cycle. However, humidity and temperature are important factors in their development, hence, they are restricted to tropical and subtropical regions with temperatures > 15.6 °C for at least 3 months of the year [16]. The sand fly life-cycle comprises four major stages: eggs, larvae, pupae and adults. Although field-based studies may reveal some valuable information on sand flies, vital information like vector competence, physiology, food preferences, parasite-vector-host relationships, insecticidal screening and xenodiagnoses studies cannot be assessed solely through field-based observations. Therefore, rearing sand flies under closed/confined laboratory conditions is essential. The techniques for establishing and maintaining sand fly colonies in different settings and regions have been well described and documented in the literature [6, 8, 17–19]. Currently, there are 90 colonies representing 21 distinct species of phlebotomine sand flies in 35 laboratories located in 18 countries worldwide [6].

Most reports on laboratory colonies of arthropod vectors are limited to information about the geographical origin of the arthropod, laboratory maintenance conditions and the necessary equipment [6–9]. However, sand fly colonies are prone to infestation by mites, ascogregarines, pathogenic bacteria and fungi that can negatively impact colony productivity [20]. In particular, ascogregarines are often associated with laboratory sand fly colonies, and high parasitemia within colonies has been shown to reduce longevity, fecundity and severe declines in the colony population [6]. The taxonomy, life-cycles, host specificity and pathogenicity of ascogregarines have been well-documented [21]. The removal of dead adults from the oviposition pots either with a vacuum aspirator or forceps and washing the eggs with 1% sodium hypochlorite to remove the oocysts from the eggshell has proven to be an appropriate procedure to reduce parasitemia levels of ascogregarines [22]. This procedure has been part of the authors’ respective insectary weekly care and maintenance activity. Some bacteria acquired by larvae from larval food may be beneficial to the fly [23] or detrimental to the fly (may cause premature death) and are transmitted transstadially to adults [24]. However, none of these microorganisms (ascogregarines, pathogenic bacteria and fungi) reached levels of infestations to cause a decline in productivity in any of the colonies in our respective insectaries. Rather, a high infestation of mites was the major challenge we faced, which heavily impacted the productivity of our colonies.

Mites are small (usually < 1 mm in length) arthropods, belonging to the class Arachnida. Their life-cycle includes the development of six-legged larvae to eight-legged nymphs, which may have from one to three nymph instars, to eight-legged adults [25]. Mite life-cycles may be completed in 8 days to 4 weeks. While reports on the diverse micro- and macro-symbionts and other co-inhabitants occurring in established research colonies of arthropod vectors are available, there is little information derived from an in-depth analysis of the mite-hematophagous arthropod relationship in a closed colony. Here, we describe *Tyrophagus* sp. and *Stratiolaelaps scimitus* mites associated with sand fly colonies in two different laboratories, one at the Laboratory of Malaria and Vector Research (LMVR), National Institute of Allergy and Infectious Diseases–National Institutes of Health (NIAID-NIH;  Rockville, MD, USA) and the other at the University of Calgary (UCalgary, Calgary, AB, Canada). We further discuss the nature of this association, its impact on sand fly production and the control measures adopted in both laboratories.

## Methods

### Mite infestation of long-term laboratory colonies of sand flies

**Fig. 1:**
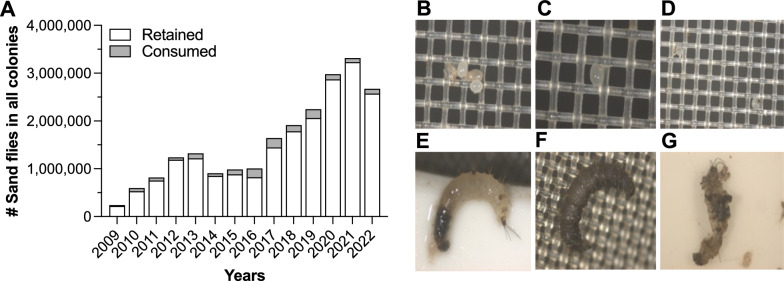
Number of sand flies in all colonies at LMVR-NIH, and photomicrographs showing mite eggs and their detrimental interactions with sand fly larvae in rearing pots. **a** Number of sand flies retained in colonies versus those consumed in research projects from 2009—2022. **b**, **c**, **d** Mite eggs in sand fly larval pot lids (300-μm screen). **e** Sand fly at fourth-instar larval stage bitten by an adult mite in search of hemolymph. **f** Necrotic sand fly at fourth-instar larval stage with progressive dark larval cuticle due to mite attack. **g** Dead sand fly at fourth-instar larval stage. LMVR-NIH, Laboratory of Malaria and Vector Research–National Institutes of Health

At the LMVR-NIH, the number of sand flies retained to maintain colonies versus those consumed in different laboratory projects in long-term laboratory colonies was recorded (Fig. 1a). From 2009 to 2013, the number of reared sand flies grew exponentially annually; however, in early 2013 after an attempt to colonize *Phlebotomus argentipes* from a mite-infested source, the overall growth of the colonies experienced a sudden 31.6% decrease. This infestation heavily impacted the small-sized colonies of *Phlebotomus perniciosus* and *Phlebotomus sergenti*, which persisted for 3 years (2014 to 2016) until the mites could be controlled and colonies regained momentum with 54,000 (2014), 104,274 (2015), and 180,147 (2016) sand flies, respectively (Fig. 1a). For the first time in the sand fly literature, we were able to track, photograph and videotape mite development under conditions of detrimental larval interactions in sand fly larval pots (Fig. 1b-g; Additional file 1: Video S1). Once the sand fly larva is dead in the presence of organic matter (humus-like larval food), it decomposes and disappears quickly, allowing only mites to multiply and infest the larval pots.

**Fig. 2:**
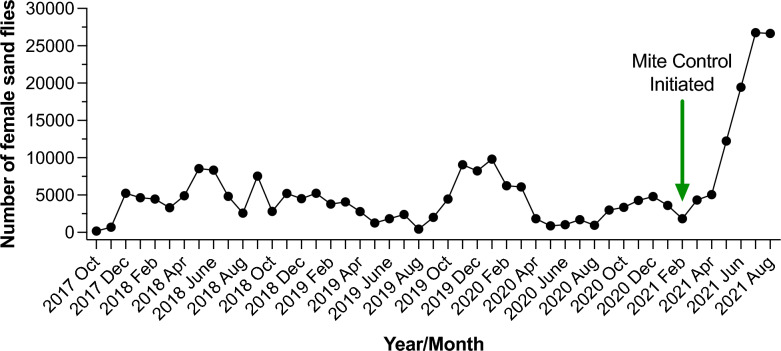
Graph showing a near crash of a working laboratory colony of *Lutzomyia longipalpis* due to mite infestations and subsequent progressive recovery after meticulous changes in laboratory procedures and mite control were initiated to contain the mite infestations

At UCalgary, the *Lutzomyia longipalpis* laboratory colony was established in October 2017 with flies from Walter Reed Army Institute of Research (WRAIR; Silver Spring, MD, USA). Although these flies were contaminated with mites, we were able to establish the colony successfully. To enhance colony productivity, we supplemented the colonies with flies from LMVR-NIH in August 2018. These flies came with unusual mite populations that differed from those of the mites from WRAIR, and within a short period, the introduced mites had spread throughout the colony with virtually no larval pots remaining mite free (Fig. 2). The mites also invaded the *Phlebotomus duboscqi* colony. The overall growth of the colony fell drastically by 30.9%, which we suspected was due to the high numbers of mite infestations. The sand fly numbers subsequently recovered to some extent between September 2019 and March 2020 (Fig. 2), in response to standard mite control measures, such as daily removal of dead individuals, ovipots checked daily and mite removal [6]. Unsurprisingly, the mite numbers increased again during the COVID-19 pandemic lockdown period (March–June 2020) because we were not able to meticulously control the mites due to COVID-19 safety restrictions, which led to mite proliferation in the rearing pots and affected the sand fly productivity. At that point, most of our attempts to control the mite infestations failed and were too time-consuming to be applied on a large scale. The decimation of colony numbers and productivity could not be reduced (Fig. 2).

### Mite collection and identification

**Fig. 3:**
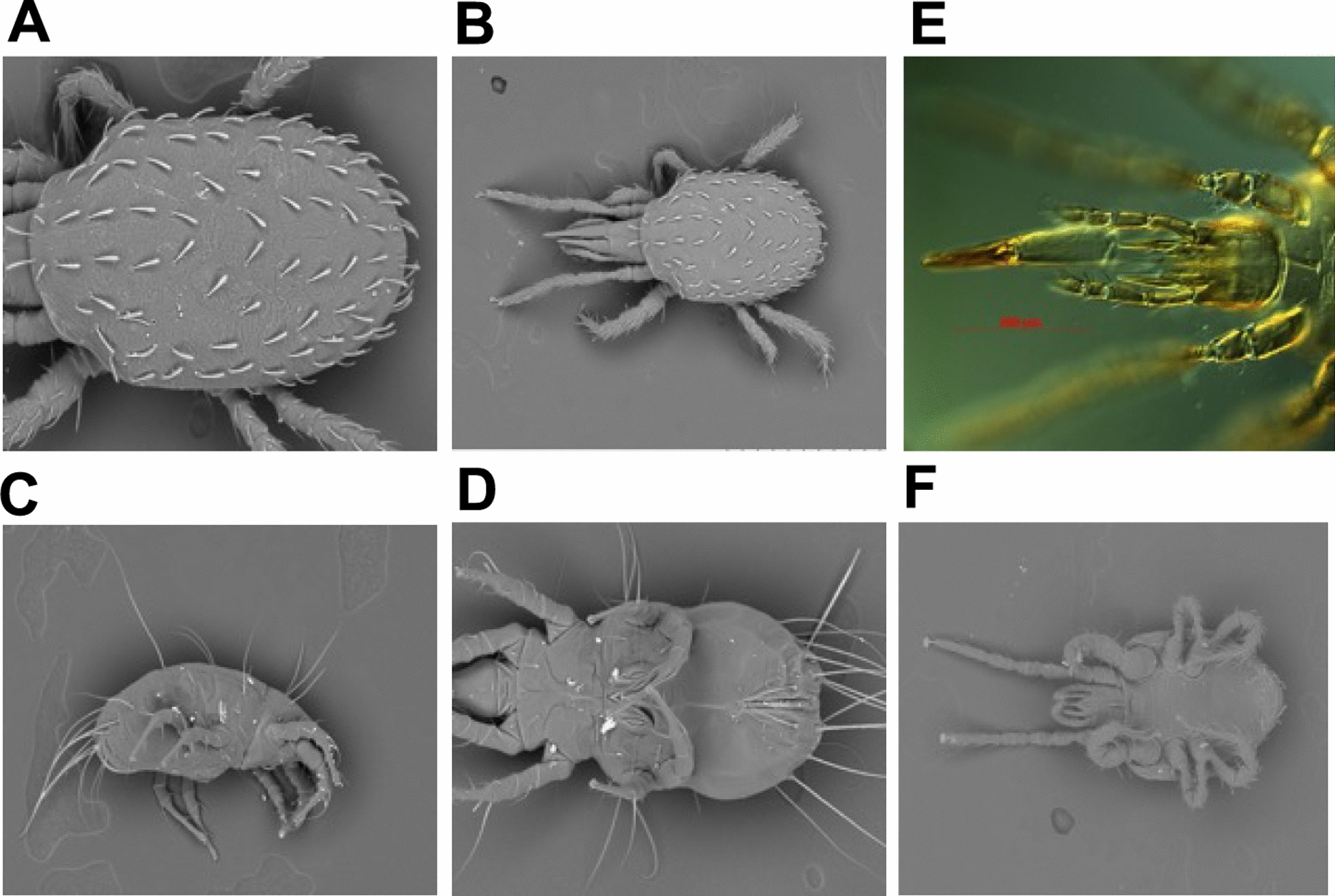
Photomicrographs of *Stratiolaelaps scimitus* mites from the sand fly colony. **a** Female dorsal idiosoma, **b** female dorsal overview, **c** female lateral overview, **d** female ventral overview, **e** female proboscis, **f** male lateral overview

Accurate knowledge of the identity of arthropod species is essential for a better understanding of their distribution and control measures needed [26]. To identify the mite species that overwhelmed the sand fly colonies at the LMVR-NIH, mites were collected from the larval pots, stored in 70% ethanol and shipped to a mite laboratory under the direction of Dr. Ron Ochoa at the U.S. Department of Agriculture (USDA), Agriculture Research Services (Beltsville, MD, USA) for proper clearing, mounting and identification. Upon identification as *S. scimitus* (Mesostigmata: Laelapidae), the mites were photographed with a scanning electron microscope (Fig. 3), and specimens were permanently preserved on glass slides stored together with the vast mite acarological collection maintained at the USDA Beltsville center.

The different mites associated with sand fly colonies at UCalgary were morphologically identified using taxonomic keys [27, 28] to genus level as *Tyrophagus* sp. (Sarcoptiformes: Acaridae); those introduced from LMVR-NIH were identified to a species level as *Stratiolaelaps scimitus*.

## Results

### Laboratory colonization of sand flies

The sand fly section of LMVR at the NIAID-NIH has a long-lasting history of mass-rearing several sand fly species of medical importance. The laboratory colonies of the species maintained at this location were initiated with field-collected specimens and imported into the USA as egg masses from their respective countries (Table 1).

**Table 1:** Sand fly species colonies maintained at the Laboratory of Malaria and Vector Research, National Institutes of Health and the University of Calgary

Species	Year colony established	Origin: city/county/state/country
LMVR-NIH		
* Lutzomyia longipalpis*	2007	Jacobina, Bahia State, Brazil
* Lutzomyia longipalpis*	2010	Cavunge, Bahia State, Brazil
* Phlebotomus argentipes*	2021	Aurangabad, Maharashtra State, India
* Phlebotomus duboscqi*	2009	Baraoueli District, Mail
* Phlebotomus papatasi*	1997	Jordan Valley, Jordan
* Phlebotomus perniciosus*	2009	Italy
* Phlebotomus sergenti*	2009	Israel
UCalgary		
* Lutzomyia longipalpis*	2017	Jacobina, Bahia state, Brazil
* Phlebotomus duboscqi*	2017	Baraoueli District, Mail

*LMVR-NIH* Laboratory of Malaria and Vector Research, National Institutes of Health, Rockville, MD, USA,* UCalgary* University of Calgary, Calgary, AB, Canada

In October 2017, we established *Lu. longipalpis* and *P. duboscqi* sand fly colonies (Table 1) at UCalgary, adding to the inventory of sand fly colonies in the world. The laboratory colonies of these species were initiated with flies from WRAIR via the Biodefense and Emerging Infections Research Resources Repository (BEI Resources; Bethesda, MD, USA). To date, the UCalgary facility is the only one in Canada that maintains insect vectors to study the transmission of infectious diseases employing animal models.

Rearing conditions have a direct and often irreversible effect on adult traits of insects [6, 29, 30]. In general, sand fly colonies are maintained in a room with controlled humidity and temperature or in an incubator at 25—28 °C and 70–80% relative humidity (RH). Different laboratories rear sand flies under different light:dark photoperiods, such as 14:10 h, 12:12 h or 16:8 h [6]. Optimum temperature and RH are important factors in the development of sand flies and may vary depending on the species or life stages. Further, the quality of larval food is a critical factor during early larval stages. Larval food preparations have been extensively reported in the literature [6, 8, 17–19]. Larval pots should be checked for food at least twice per week, and the food should be replenished according to the number of larvae and their size. Excessive food leads to fungal growth and mite infestations whereas underfeeding results in cannibalism and unsynchronous development [6, 8].

### *Tyrophagus* sp. and* S. scimitus* control measures

#### Mite control measures at LMVR-NIH

At the LMVR-NIH, as soon as the mites were detected at detrimental numbers, several methods were attempted to contain or even eradicate their quick spread. Washing the sand fly eggs in benzoyl peroxide (Luperox A98; manufacturer part number [MPN] 179981; Sigma-Aldrich, St. Louis, MO, USA) and hydrogen peroxide solution at 2—5% (MPN H1009; Sigma-Aldrich) proved to be ineffective. Similarly, washing in Dicofol 4-E (Pestanal; MPN 36677; Sigma-Aldrich) miticide equivalent to Kelthane MF and benzyl benzoate USP grade (www.MedLabsupplies.com) was extremely toxic even when highly diluted, preventing sand fly eggs from hatching. Hypertonic and hypotonic solutions, commonly available in any laboratory setting (e.g. NaCl ranging from 100 to 600 mM) were equally ineffective in killing mite eggs.

The commercially available miticide (Genesee Scientific Corp., Morrisville, NC, USA; product number 59–130) for *Tyrophagus* sp. mites showed non-toxicity to the sand fly eggs and was relatively successful. To test this miticide, sand fly carcasses were removed by vacuum aspiration under a microscope, eggs were washed [6] and the foam sprayer that accompanied the miticide was added to pre-soak the eggs for 10–15 min. After the foam settled, more miticide was added until all eggs were completely submerged in the retaining sieve.

Cleaned and decontaminated eggs were returned to new larval pots (catalog number 2117–0500; Nalgene Thermo Fisher Scientific, Waltham, MA, USA). The larval pots were stored in sealed tight plastic trays (catalog number 3507; Rubber-Maid, Atlanta, GA, USA) and placed in Percival incubators (model I-36VL, catalog number 50220; Percival Scientific Inc., Perry, IA, USA) maintained at 27 °C/65% RH/12:12-h photoperiod, cleaned and decontaminated thoroughly with the same miticide used previously. The sand fly walk-in chambers, which were custom-made by Conviron (www.Conviron.com) were power washed, and 100% Castor oil from Nature’s oil brand (www.Naturesoil.com) was applied to the base boards, benches, plastic racks and surfaces. Sticky mats (model H-1567W; Uline, Pleasant Prairie, WI, USA) were laid down before entering and exiting the sand fly walk-in chambers.

The lids of larval pots were replaced with plankton netting with 300-μm openings (Bioquip model 7293B; www.Bioquip.com) to replace old lids with small 1-inch vented snap cap lids in panel plugs (product 9688K688; McMaster-Carr Supply Co., Elmhurst, IL, USA) to reduce mite migration and cross contamination between pots. However, this method was only used for a relatively short period for the pots of *Lu. longipalpis* because they tend to produce more moisture due to larvae breathing. Pots were not autoclaved to prevent plaster breakage but instead kept in the freezer at − 20 °C for ≥ 72 h before larvae excreta were double bagged for proper disposal in Medical Pathological Waste (MPW) boxes.

For breeding, adult sand flies are kept in acrylic cages custom-made by Precision Plastics (Beltsville, MD, USA) which were placed on top of inverted plastic boxes coated with Castor oil to prevent cross-contamination between cages from migrating mites. After one cycle of blood-feeding and the capture of blood-fed female flies into ovipots for egg laying, cages were thoroughly washed with a soft sponge soaked in Alconox (Aloncox Inc., White Plains, NY, USA) and decontaminated with 70% ethanol followed by several rinses of distilled water before being reused. Sugar traps consisting of small amounts of 70% Karo® dark corn syrup solution poured into Petri dishes were placed around each chamber’s perimeter and inside incubators to promptly trap migrating mites. These traps were checked and replaced daily to avoid mold growth and to remove as many roaming mites as possible.

**Fig. 4:**
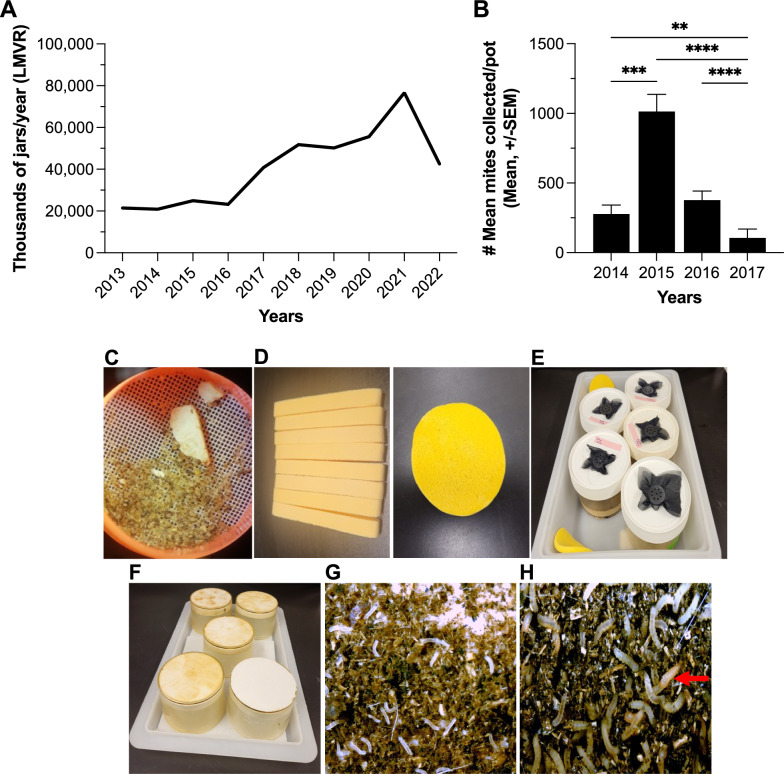
Larval production following meticulous mite control and changes initiated to reduce infestations. **a** Number of larval pots/jars maintained at the LMVR-NIH from 2013 to 2022. **b** Annual mite counts from 10 randomly chosen pupal pots throughout colony infestation and post-treatment with miticide (LMVR-NIH), Kruskal Wallis Test with Dunn's multiple comparisons. **< 0.0057, ***= 0.0001 and ****< 0.0001. **c** Collection of adult *Tyrophagus* sp., *Lu. longipalpis* adult carcasses and plaster (Home Depot Inc., Atlanta, GA, USA) debris in a 300-μm cell strainer. **d** Fiber polymer absorbent facial sponges (BWXXR), which come in compressed sticks (left), open in water to round (right). **e** Wet fiber polymer absorbent facial sponges in rearing tray for maintaining humidity, and perforated lids of larval pots covered with a fine black gauze top secured with an open plastic cap (with a hole cut in the middle). **f** Washed-rearing pots stored upside-down in a clean tray lid until use. **g** Larval pots containing robust and healthy first- and second-instar larvae. **h** Third- and fourth-instar larvae and pupae (red arrow) of *Lu. longipalpis* sand flies (UCalgary). NMVR–NIH, Laboratory of Malaria and Vector Research, National Institutes of Health, Rockville, MD, USA. UCalgary, University of Calgary, Calgary, AB, Canada

In 2013, we decided to start counting the number of larval pots twice a week for each colony as part of our rearing routine. Although absolute numbers do not express the real quality of larval pots, we needed an additional method to ensure adequate colony growth. This was needed to safely allow us to determine the number of larval/pupal pots that we could ship to colleagues and for use in out-of-country projects without compromising our colonies. Coincidentally, because the pot count system was implemented the year before the mite infestation, we were able to account for the decrease in the number of pots during that period and thereafter when sand fly numbers were restored (Fig. 4a). The data show that in the year following the severe infestation, pot numbers increased significantly (75.8%) until the end of 2021 when we decided to limit the number of pots due to limiting factors such as manpower, laboratory budget, the numbers needed to meet the demand for projects and the high consumption of larval food (custom made in the laboratory). With the implementation of pre-soaking sand fly eggs in miticide along with all other methods described herein, we were able to significantly reduce the infestation over 3 years (Fig. 4b). Pots with emerging flies were kept for a short term—half of the regular time—just until the bulk of flies emerged in order to avoid creating a breeding place for more mites to thrive, even if this procedure resulted in sacrificing some flies.

#### Mite control measures at UCalgary

At UCalgary, we employed measures to contain the mite infestations to a minimum level. To aid in the collection of the mites, yeast traps were placed in plastic trays (product 3507; Rubber-Maid Inc.) containing larval pots [31]; however, no significant success was ever achieved. Sand fly larval food and rabbit dung were frozen at − 20 °C for 48 h and > 1 month, respectively, as a potential means to eliminate infesting mites but, ultimately, mites still emerged from both the larval food and the rabbit dung. This observation was consistent with other reports that mite eggs are not killed by freezing, even after several hours at − 18 °C [32], although it is not known why some eggs may survive these temperatures. Further, larvae were fed with a mixture of 66% larval food and 33% organic soybean flour (Bob’s Red Mill, Milwaukie, OR, USA) to inhibit mite development in the larval pots, and/or washed sand fly eggs were kept in oviposition pots sprayed with 0.5% soybean oil to interfere with the respiratory activity of the mites by blocking their spiracles. These attempts were relatively successful, although we were cautious of the utility of organic soybean flour since some sand fly species may be sensitive to potential changes in either the fungal profile or altered sand fly microbiota that may accompany a change in food composition [6].

After sand fly oviposition, carcasses and mites were removed from the ovipot with a vacuum pipette aspirator. To ensure that all mites were removed, we added a step with metal strainer (mesh size 200–300 μm) to the sand fly egg washing protocol for the insectary, with the aim to aid the separation of fly and mite eggs (Fig. 4c). However, the filtration through mesh was insufficient to remove mite eggs. Therefore, to improve fly and mite egg separation, we tested sucrose gradient centrifugation (2 ml; gradient from top to bottom of 3%, 13%, 24%, 35%, 54%) followed by centrifugation for 20 min at 800 *g* with slow acceleration and deceleration stages and spinning at 4 °C [33]. Unfortunately, the specific gravity of mite eggs could not be evaluated using this method due to the extreme transparency of the eggs.

However, implementation of the above treatments/efforts were not effective in eliminating or reducing the mite population, and the survival of the colonies was in jeopardy and heading toward a “colony crash.” In February 2021, we meticulously made some changes in our insectary, which not only prevented the colony from crashing but progressively increased sand fly production to an unprecedented high number of 26,759 flies in July 2021 (Fig. 2). In the following text, we describe in detail some of the measures we took.

Sand fly-rearing incubators were shut down and thoroughly cleaned with a sponge and water (no soap solution or bleach) and sanitized with 70% ethanol (not isopropanol), including sanitization of the built-in humidifier at the base of the incubators. The larval food compositing chamber was cleaned with a sponge and water and sanitized with 70% ethanol. Ideally, keeping the food compositing chamber out of the insectary, where applicable, is encouraged. Working benchtops and entire insectary surroundings were adequately cleaned with water and soap. Ovipot trays were cleaned with running tap water within the sink installed in the insectary.

Mites tend to accumulate on wet sponges (for maintaining humidity) kept in the ovipot trays. These regular kitchen sponges not only become frayed or torn after long-term use but if not washed regularly lead to mold and fungal growth. As part of our efforts to reduce cyclic mite infestation, we replaced the regular kitchen sponges with fiber polymer absorbent facial sponges (BWXXR), which come in compressed sticks, open in water to form a round shape, are long-lasting and are commercially available (Fig. 4d, e). The old, perforated lids of larval pots initially covered with a fine white gauze top secured with an open plastic cap (with a hole cut in the middle) were replaced with a fine ‘black gauze’ (Fig. 4e) to allow better visualization of mites by the naked eye. This change greatly improved the efficiency of mite removal and reduced mite migration and cross-contamination between pots. The old rearing pot Plaster of Paris from Home Depot Inc. was replaced with new dental plaster (K-Dental Inc., Markham, ON, Canada). In addition, before larvae feeding and mite removal, the workbench top was sprayed with 70% ethanol to create an ethanol pool. The surface of each pot was brushed off with a paper towel to remove mites within the area sprayed with ethanol. The pots were opened, and the lids knocked on top of the sprayed ethanol to dislodge mites into the ethanol pool. These steps were incorporated into our insectary standard of operating procedure (SOP) because they yielded positive outcomes in terms of reducing mite infestations in our sand fly colonies. After adult emergence, pots were frozen at - 20 °C for ≥ 3 days and then washed in hot water with a sponge (no detergent); the plaster layer in the bottom of the pot was scrubbed with a paper towel. Pots were then stored upside-down in a clean tray until use (Fig. 4f). To prevent cyclic contamination, trays, nets, cages and all transfer apparatus were regularly cleaned and sanitized with 70% ethanol. By applying these measures, we were able to fully restore our colony productivity within 3 months (Fig. 2), and the larvae were robust and “healthy” (Fig. 4g, h).

## Discussion

Mites are hitch-hikers, often attach themselves to an insect or other animal, get transported to another place (phoretic), reproduce rapidly and are quick to colonize new habitats [34]. They are highly adaptable, ubiquitous and capable of living in various habitats. Phlebotomine sand flies harbor a rich fauna of mites, which may be phoretic, parasitic or both. At the present time, 15 different families, 16 genera, and 21 species of mites have been recorded on 39 species of the adult sand fly exoskeleton [35, 36].

*Tyrophagus* is a ubiquitous mite, commonly found in stored food products and decaying organic matter. Larval pots, food composting chambers and larval food are easily contaminated by *Tyrophagus* mites [8]. We also found the emergence of *Tyrophagus* mites from rabbit dung stored at − 20 °C for months outside the insectary that was used in the preparation of sand fly larval food, suggesting another source of mite infestation. These mites, when present in large quantities, can easily damage the colony by eating larval food and secreting metabolites that stop larval development [8]. *Tyrophagus* mites have also been reported infesting various laboratory colonies of insects, such as *Aedes* sp. mosquitoes [7], Africanized honeybees [37], *Drosophila* sp. [38] and soft ticks [9]. On the other hand, *S. scimitus* is a predatory mite that feeds on fungus gnats, thrips pupae and other small insects in the soil. *Tyrophagus* and *Stratiolaelaps* mites flourish well under the same developmental conditions of 25–28 °C and 70–80% RH as the sand fly colonies, resulting in the rapid growth of mite infestations in these colonies. *Amblyseilus*, a predatory mite, has been reported to be a good biological control of the *Tyrophagus*, is not dangerous even to first-instar sand fly larvae and is commercially available [8]. The source of *S. scimitus* and its impact on different sand fly species colonies needs to be further investigated.

Although mites are regularly observed in the sand fly colonies, their competition for larval food and the adverse effect of their activities in decimating colony numbers and productivity, if not controlled, could negatively impact vector-borne disease studies. In addition, uncontrolled mite infestation adds to the already labor-intensive nature of sand fly colony maintenance and could further discourage entomologists or scientists from colonizing sand flies in the laboratory.

Based on our experiences, the following points should be considered:

1. When receiving sand flies from any source, always keep them segregated inside a quarantine incubator away from the main colonies for several generations, or until fully satisfied that they do not represent a threat to the existing colonies. All colony-rearing materials should also be segregated and never be placed near or shared with healthy colonies.
2. All sand fly colonies are equally susceptible to mites. However, it should be noted that the longer the larval life-cycle, the greater and more severe the infestation in the pots; this was the case of *P. duboscqi* and *Phlebotomus papatasi* with 40–45 days from eggs to adults
3. Miticide (Genesee Scientific) was effective in reducing the mite population; however, it should not be implemented as the sole method to contain a severe mite infestation or be equally considered a “magic bullet.” All methods combined resulted in a reduction in the mite population in our respective colonies over time. In addition, despite its continuous use for a prolonged period, the miticide did not influence the experiments related to sand fly infection with *Leishmania* or affect the life-cycle of sand flies in the colony at LMVR-NIH.
4. Complete mite eradication is impossible to achieve, even in closed sand fly colonies, because there are factors beyond the laboratory's control, such as the source of rabbit dung/feces for larval food preparation and hosts utilized in colony blood-feeding, such as chickens (*Gallus gallus*), which are notorious for dispersing mites in natural habitats. We do not use autoclaved larval food because we infect sand flies with *Leishmania* parasites in our laboratories; therefore, preservation of natural microbiota is essential to promote strong and reproducible infections. In addition, it has been reported that autoclaved larval food/diet reduces larval productivity, possibly due to excessive mold growth when used to feed the colony [39]. This excessive growth is likely to be due to the absence of bacteria, as the excessive mold growth was reduced and larval productivity was restored by the prior addition of a small amount of supernatant liquid from an aqueous slurry of rabbit dung (a source of bacterial community’s restoration) to the autoclaved larval diet [39]. That larval food alone would be sufficient to generate mites is likely to be inaccurate. Several additional factors need to be at play to make a mite population proliferate and spread within the rearing pots, including optimum RH and temperature.
5. The overall level of mite infestation (such as low, moderate, or high) in larval pots should be incorporated into the Weekly Log for the Sand Fly Insectary. Although this is qualitative and/or descriptive step, tracking the levels of infestation in the larval pots at least twice per week, especially during larvae feeding and mite control, has helped us to properly monitor the mite infestation level in our colonies and prevent it from reaching the high levels that are detrimental to sand fly colonies.

## Conclusions

Mite infestations are almost impossible to avoid. Because of their small size (approx. 0.5 mm) mite populations residing in fringe habitats often go unnoticed. It is not until these populations encounter ideal humidity and temperature conditions, as well as stable food sources that their populations become large enough to be noticed and inhibit colony stability and growth. Mites existing in peripheral harborage areas or undetected in at-risk surfaces may cause cyclic infestations that are both time-consuming and difficult to control, impeding research studies; ultimately, they may lead to a sand fly “colony crash”. Taken together, we report a case of detrimental laboratory interactions between sand flies and *Tyrophagus* and *Stratiolaelaps* mites and provide guidelines for overcoming severe mite infestations in sand fly colonies. No single measure can adequately reduce mite infestations in the laboratory insect vector colonies. However, good housekeeping and cleanliness in the insectary cannot be over-emphasized and are paramount in reducing cyclic mite infestations.

### Supplementary Information

**Additional file 1: Video S1.** Colonization of adult mites on sand fly larval pot.

## Data Availability

All data generated or analyzed during this study are included in this published article. *Stratiolaelaps scimitus* (Mesostigmata: Laelapidae) specimens are permanently preserved on glass slides stored along with the vast mite acarological collection maintained at the USDA Beltsville center, MD, USA.

## Abbreviations

BEI Resources: Biodefense and Emerging Infectious Research Resources Repository
LMVR: Laboratory of Malaria and Vector Research
MPW: Medical pathological waste
NIAID: National Institute of Allergy and Infectious Diseases
NIH: National Institutes of Health
UCalgary: University of Calgary
USDA: United States Department of Agriculture
WRAIR: Walter Reed Army Institute of Research
